# A perspective from the EU: unintended genetic changes in plants caused by NGT—their relevance for a comprehensive molecular characterisation and risk assessment

**DOI:** 10.3389/fbioe.2023.1276226

**Published:** 2023-10-27

**Authors:** Franziska Koller, Michael Cieslak

**Affiliations:** Fachstelle Gentechnik und Umwelt (FGU), Munich, Bavaria, Germany

**Keywords:** new genomic techniques (NGT), genetically engineered organisms, genome editing, GMO regulation, risk assessment, unintended genetic changes in NGT plants, comprehensive molecular characterisation

## Abstract

Several regions in the world are currently holding discussions in regard to the regulation of new genomic techniques (NGTs) and their application in agriculture. The European Commission, for instance, is proposing the introduction of specific regulation for NGT plants. Various questions need to be answered including e.g., the extent to which NGT-induced intended and unintended genetic modifications must be subjected to a mandatory risk assessment as part of an approval procedure. This review mostly focuses on findings in regard to unintended genetic changes that can be caused by the application of NGTs. More specifically, the review deals with the application of the nuclease CRISPR/Cas, which is currently the most important tool for developing NGT plants, and its potential to introduce double strand breaks (DSBs) at a targeted DNA sequence. For this purpose, we identified the differences in comparison to non-targeted mutagenesis methods used in conventional breeding. The review concludes that unintended genetic changes caused by NGT processes are relevant to risk assessment. Due to the technical characteristics of NGTs, the sites of the unintended changes, their genomic context and their frequency (in regard to specific sites) mean that the resulting gene combinations (intended or unintended) may be unlikely to occur with conventional methods. This, in turn, implies that the biological effects (phenotypes) can also be different and may cause risks to health and the environment. Therefore, we conclude that the assessment of intended as well as unintended genetic changes should be part of a mandatory comprehensive molecular characterisation and risk assessment of NGT plants that are meant for environmental releases or for market authorisation.

## 1 Introduction

According to EU GMO legislation (European Parliament and Council of the European Union, 2001), genetically modified organisms (GMOs) derived from “recombinant nucleic acid techniques” are to be regulated [Annex 1A, Part 1 of (European Parliament and Council of the European Union, 2001)]. As clarified by the European Court of Justice (Case C-528/16), this also applies to organisms derived from “new genomic techniques” (NGTs). The detailed risk assessment requirements are set out in Annex II of Directive 2001/18/EC (European Parliament and Council of the European Union, 2001) which was last amended in Commission Directive (EU) 2018/350 (European Commission, 2018). As introduced in the Annex (C1) of this in Commission Directive (EU) 2018/350 (European Commission, 2018), risk assessment “shall identify the intended and unintended changes resulting from the genetic modification and shall evaluate their potential to cause adverse effects on human health and on the environment.” Furthermore, Annex II of Directive 2001/18/EC (European Parliament and Council of the European Union, 2001) in its “Principles for the environmental risk assessment” also gives weight to cumulative long-term effects.

For the purposes of this review, we use the same specific terminology as Koller et al., 2023 to distinguish between several categories of GMOs belonging to plants (Koller et al., 2023): 1) EU GMO regulation refers to GMOs which have to undergo mandatory approval processes and other GMOs which are exempt from these approval processes, e.g. plants derived from physical and chemical mutagenesis. The term “genetic engineering” (GE) is used throughout the review as a synonym for those GMOs which have to undergo mandatory approval processes; and 2) the term “established genomic techniques” (EGTs) is used to distinguish “old” GE plants (derived from non-targeted insertions of transgenes) from those more recently generated using NGTs (see also (EFSA, 2022a)). It is important to understand that both these categories (EGT and NGT) refer to GMOs which have to undergo mandatory approval processes (GE) according to the current legal situation.

Our review examines whether current EU regulation must in future continue to include the risk assessment of unintended genetic changes in NGT plants. In order to come to a sufficiently reasoned conclusion, our review first provides an overview of published findings related to unintended genetic changes caused by NGT processes in plants. Further, we identify causes for unintended genetic changes to differentiate these changes from non-targeted mutations which occur in conventional breeding. Finally, we discuss the consequences for the risk assessment of single events (individual NGT organisms), and long-term accumulated effects.

## 2 Differences between genetic changes caused by NGTs and conventional breeding

In short, and as summarized also by Kawall (2019) and Koller et al. (2023), site directed nucleases (SDN), such as CRISPR/Cas (clustered regularly interspaced short palindromic repeats/CRISPR associated) (Jinek et al., 2012), are highly relevant in this context: they are designed to target specific DNA sequences in the genome to knock out gene functions (i.e. SDN-1) or to introduce specific changes of particular nucleotides (i.e. SDN-2) or whole genes (i.e. SDN-3). These methods can induce either non-specific changes (SDN-1) via non-homologous end joining (NHEJ) repair mechanisms or specific changes to nucleotide sequences (SDN-2 or SDN-3) via homologous recombination mediated by homology directed repair (HDR). The latter require an additional template. The induced changes at or around the target site can be substitutions, deletions or insertions of one or more base pairs. Depending on the specific SDN-1 or SDN-2 application, more extensive overall alterations are possible. For example, using multiplexing it is possible to target several genes simultaneously in a single application (Raitskin and Patron, 2016; Wang et al., 2016; Zetsche et al., 2017). Repeated applications of SDN-1 or SDN-2 can also be combined (Kawall et al., 2020). Changes involving the insertion of whole (cis- or trans-) genes (including gene-stacking) are also possible (SDN-3) and are mediated by the use of specific donor DNA (Sander and Joung, 2014; Eckerstorfer et al., 2019). For this review, we mostly focus on applications using CRISPR/Cas and its potential to introduce DSBs at targeted DNA sequences which is currently the most important tool for developing NGT plants (Parisi and Rodriguez, 2021). Other nucleases, such as TALENs (transcription activator-like effector nucleases) or variations of CRISPR nucleases (Parisi and Rodriguez, 2021), are also relevant, but so far of less importance for NGT in plants.

As has been shown many times [see for example (Morineau et al., 2017; Nonaka et al., 2017; Sánchez-León et al., 2018; Raffan et al., 2021)], NGTs enable the emergence of new genotypes and phenotypes to be generated in different ways and with different outcomes compared to previously used genetic engineering methods or conventional breeding (including non-targeted mutagenesis) (Eckerstorfer et al., 2019; Kawall, 2019; EFSA et al., 2021a; Kawall, 2021a; Kawall, 2021b).

In comparison to methods of conventional breeding (including non-targeted mutagenesis), NGTs can overcome the boundaries of natural genome organization: Relevant factors include repair mechanisms, gene duplications, genetic linkages and other epigenetic mechanisms [see, for example, (Lin et al., 2014; Wendel et al., 2016; Filler Hayut et al., 2017; Frigola et al., 2017; Roldan et al., 2017; Belfield et al., 2018; Huang and Li, 2018; Jones et al., 2018; Halstead et al., 2020; Monroe et al., 2022)]. By overcoming these boundaries, NGTs can make the genome much more extensively available for genetic changes (Kawall, 2019; Kawall et al., 2020).

In comparison to conventional plant breeding using non-targeted mutagenesis, the overall number of mutations is typically lower in NGT plants (Modrzejewski et al., 2020). However, due to the technical characteristics of NGTs, the sites of the mutations, their genomic context and their frequency (in regard to specific sites) can differ if compared to plants derived from conventional breeding methods. Such a non-random occurrence of mutations along the genome can therefore also be expected for the unintended genetic changes. This, in turn, means that the biological effects (phenotypes) can also be different and may cause specific risks for health and the environment.

Furthermore, it has to be considered that the processes of NGTs involve several technical steps that, in the case of plants, very often include transformation processes which are also used in EGTs. These non-targeted methods are used to introduce the nucleases into the cells [see for example (Morineau et al., 2017; Nonaka et al., 2017; Sánchez-León et al., 2018; Raffan et al., 2021)] and may lead to unintended effects in off-target regions [for example (Braatz et al., 2017), see also below].

## 3 Five categories of unintended genetic changes resulting from NGT processes with relevance to risk assessment

Unintended genetic changes resulting from NGT processes can be differentiated as those with or without the insertion of transgenes, off-target changes or on-target changes, and those which are likely to be associated with or without the production of new gene products. Furthermore, this includes the identification of smaller genetic changes versus those that involve larger parts of the genome or even complex patterns of genetic changes. While some of the “types” of genetic alteration might also be observed in conventional breeding, there may also be some differences in regard to the probability of these changes occurring at specific sites in the genome (see above). In order to differentiate between unintended genetic changes resulting from NGTs and those resulting from conventional breeding, we suggest aligning them with the following five categories.

### 3.1 Unintended genetic changes resulting from the insertion of transgenes via EGTs (off-target)

At present, NGT applications in plants are in most cases a multi-step process. For example, NGTs, such as CRISPR/Cas applications in plants, typically make use of EGT techniques, i.e. non-targeted methods, to deliver the DNA coding for the nuclease into the cells [for overview, see (Kawall et al., 2020)]. Thus, in most cases, the result of the first step of the CRISPR/Cas application is a transgenic plant which may show a broad range of unintended genetic changes, which may be different to those emerging from conventional breeding, as for example discussed by Latham et al., 2006 and more recently confirmed by Yue et al. (2022). As recently summarized by Koller et al. (2023), such effects may be linked to epigenetic regulation, the disruption of genes, position effects, open reading frames, the unintended introduction of additional genes, changes in gene expression, genomic interactions which can involve plant constituents, or plant composition and agronomic characteristics (Forsbach et al., 2003; Makarevitch et al., 2003; Windels et al., 2003; Rang et al., 2005; Gelvin, 2017; Jupe et al., 2019; Liu et al., 2019; Chu and Agapito-Tenfen, 2022; Yue et al., 2022). There are several studies showing that the problem of unintended insertion of transgenes is relevant to NGT applications in plants (Li et al., 2015; Braatz et al., 2017; Biswas et al., 2020; Michno et al., 2020) or also animals (Norris et al., 2020). Even if segregation breeding is used in plant species with sexual reproduction at the end of the multistep process, to remove the functional transgenic elements from the plant genome, unintended genetic changes may still remain in the genome unnoticed.

### 3.2 Unintended insertion of transgenes with NGT processes

As several publications show, DSBs caused by CRISPR/Cas interventions are associated with the insertion of transgenes, especially at the target site or elsewhere in the genome. These on-target and off-target effects often include the integration of DNA from vector DNA derived from transformation processes, where, for example, fragments of the transgenes were unexpectedly integrated (Li et al., 2015; Andersson et al., 2017; Braatz et al., 2017; Sánchez-León et al., 2018; Zhang et al., 2018; Biswas et al., 2020).

Also in animal cells, it was found that unintentionally inserted foreign DNA fragments may originate from the vector construct (Norris et al., 2020). In some cases, in mammalian cells, inserted additional DNA taken up from the growth medium were also found (Ono et al., 2019). Overall, the CRISPR/Cas9 system has been confirmed to have a high frequency of unintended integration of additional DNA into the target sites (Lee et al., 2019; Yang et al., 2022).

Research is underway to develop transgene free delivery of the CRISPR/Cas molecules into the plant cells [see for example (Banakar et al., 2019; Kocsisova and Coneva, 2023)]. However, questions remain upon their application in practice [see for example (Kawall et al., 2020)]. Therefore, we assume that unintended insertion of transgenes will remain a challenge in future.

### 3.3 Unintended genetic changes without the insertion of transgenes (on-target and off-target)

Various unintended genetic changes resulting from CRISPR/Cas applications have been described for plants. These include off-target DNA cleavage, repetitive unit deletion, indels of various sizes, larger structural changes in the targeted genomic region (with and without the insertion of transgenes) (Zhang et al., 2014; Kapahnke et al., 2016; Wolt et al., 2016; Braatz et al., 2017; Kapusi et al., 2017; Lalonde et al., 2017; Sharpe and Cooper, 2017; Kosicki et al., 2018; Chakrabarti et al., 2019; Biswas et al., 2020; Burgio and Teboul, 2020; Kawall et al., 2020; Manghwar et al., 2020; Michno et al., 2020; Molla and Yang, 2020; Skryabin et al., 2020; Liu et al., 2021; Yang et al., 2022; Samach et al., 2023a).

Although some of these “types” of genetic alteration might also be observed in conventional breeding (EFSA, 2020), they differ in terms of their likelihood of occurring at specific sites in the genome. Therefore, these effects can not be generally equated to those emerging from conventional breeding.

For example, larger structural genomic changes, such as translocations, deletions, duplications, inversions and scrambling of chromosomal sequences, can occur in or near the targeted genomic region which would otherwise be unlikely to occur [see e.g., (Hahn and Nekrasov, 2019)]. It should be considered that especially so-called bystander deletions and complex rearrangements in neighboring on-target sequences (EFSA et al., 2021a) may be difficult to detect (Simeonov et al., 2019).

It is known that the nucleases rather recognize and target specific DNA sequences of a particular length rather than functional genetic elements at specific genomic sites (Ahloowalia and Maluszynski, 2001; Höijer et al., 2022). Therefore, the CRISPR/Cas machinery has a potential to bind not only to the targeted regions, but also to additional off-target regions that share similarity–within a given mismatch tolerance–to the target DNA sequences. Accordingly, research is underway that tries to improve to increase the on-target efficiency and mitigate the off-target impact on intended genome-editing outcomes [such as (Wolt et al., 2016; Manghwar et al., 2020)]. However, previous studies focussing on these unintended genetic changes (Modrzejewski et al., 2019; 2020) identified gaps in the methodology such as studies being very heterogeneous in their structure and design, as well as the number of published data. Therefore, it looks like off-target effects will remain a challenge at least for the near future.

Since many of these undesirable effects as described above are often caused by DSBs introduced by the nuclease, other methods are under development that are purposed to introduce genetic changes without DSBs, especially in the area of human medicine such as base editing (Anzalone et al., 2020). These methods are also known to cause unintended genetic changes throughout the genome which requires in depth molecular characterisation and risk assessment (Rao et al., 2023). However, since these methods, so far, do not play a major role in NGT plants, they are not discussed in this review.

### 3.4 Chromothripsis-like effects

Chromothripsis is a genetic phenomenon where possibly hundreds of clustered chromosomal rearrangements can happen in a single catastrophic event. In mammals (including humans), the phenomenon is associated with cancer and congenital diseases. Available publications (Leibowitz et al., 2021; Samach et al., 2023a; de Groot et al., 2023) show that biotechnological mutagens, such as nucleases that cause a DSB in the DNA, are a likely cause of chromothripsis-like effects. According to de Groot et al. (2023), in cases where DSBs are not quickly resolved, they can be involved in rearrangements with other parts of the genome involving one or a few chromosomes. The process can be associated with deletions, insertions, inversions, duplications and double-minute formation.

It has been known that CRISPR/Cas applications strongly increase the likelihood of chromothripsis occurring in mammalian cells (Leibowitz et al., 2021; Amendola et al., 2022). Just recently, these effects were also reported in plants by Samach et al. (2023a). They identified whole chromosome losses as well as major chromosomal rearrangements, including the loss of large fragments, inversions, translocations and somatic crossovers associated with CRISPR/Cas-induced DSBs.

DSBs also may occur if, for example, plant cells are exposed to high dosage of radiation (non-targeted mutagenesis) (EFSA et al., 2021b). However, NGTs may impact the probability of chromothripsis occurring at specific genomic sites with a higher likelihood and therefore, its biological effects may depend on the genomic regions that are targeted by the processes of NGTs. For example, in plants with many copies of the targeted genes [see, for example, (Sánchez-León et al., 2018)], CRISPR/Cas is likely to cause several DSBs simultaneously in a specific pattern. Similarly, many DSBs can be caused by targeting several genes in parallel [“multiplexing”, see (Zsögön et al., 2018)]. Furthermore, the CRISPR/Cas machinery can interfere with the repair mechanisms in the cells, preventing them from restoring the original gene functions and stopping the cells from rapidly resolving the DSB [see (Kawall, 2019)].

These findings make it plausible that DSBs and chromothripsis-like effects caused by biotechnological mutagens (nucleases) should not generally be equated with those of non-targeted physical-chemical mutagens.

### 3.5 Unintended genetic changes that may cause the formation of new gene products (without insertion of transgenes)

The use of CRISPR/Cas gene scissors can induce various changes at the target sites. The targeted site (or also off-target sites) can be altered in such a way that no more mRNAs are formed, thus preventing the formation of the corresponding protein. However, new mRNAs can also be unintentionally formed, and thus cause new proteins to emerge.

For example, the changes introduced by the nucleases can lead to an effect called exon skipping. In exon skipping, mRNAs can be assembled differently than planned even if the intended changes are induced at the target site. This can lead to the formation of shortened mRNAs. The resulting proteins are then also shorter, but can still carry out functions in the cell. The effects of exon skipping were described in mammalian cells (Kapahnke et al., 2016; Mou et al., 2017) as well as in plant cells (Ramírez-Sánchez et al., 2016). In this context, also frameshift mutations are described. They cause a shift in the reading frame of a DNA sequence which may go along with change in the gene function (Lalonde et al., 2017).

As a result of exon skipping and frameshift mutations, new mRNAs and proteins, or also non-coding RNAs (ncRNA) with effects on gene regulation, can be formed and fulfill new functions in cell metabolism (Kapahnke et al., 2016; Lalonde et al., 2017; Mou et al., 2017; Tuladhar et al., 2019; Jia et al., 2022). For example, effects caused by knocking out of 35 gene copies in wheat (Sánchez-León et al., 2018) were discussed by EFSA et al. (2021a) as a potential cause for the occurrence of peptide fragments that could play a role in the inflammatory cascade (see also below). Frameshift mutations may play a significant role in the emergence of such fragmented peptides.

Again, since these unintended genetic changes may not occur randomly across the genome, its biological effects may depend on the genomic regions that are targeted by the processes of NGT. These effects can not be generally equated to those emerging from conventional breeding.

## 4 Consequences for a comprehensive molecular characterisation and risk assessment of single events

According to EU regulation as cited above (European Parliament and Council of the European Union, 2001; European Commission, 2018), it has to be taken into account that unintended genetic changes “can have either direct or indirect, and either immediate or delayed effects on human health and on the environment.” Therefore, the risk assessment “shall identify the intended and unintended changes resulting from the genetic modification and shall evaluate their potential to cause adverse effects on human health and on the environment.”

Based on the various findings regarding unintended genetic effects that NGTs can cause, it does not appear possible to predict or control their occurrence and associated effects for specific events. As shown, unintended genetic changes can affect large sections of chromosomes and result in the emergence of unintended gene products. Since these unintended genetic changes may not occur randomly across the genome, its biological effects may depend on the genomic regions that are targeted by the processes of NGTs and therefore are also relevant for risk assessment.

It is only afterwards through applying methods, e.g. whole genome sequencing (WGS) and other methods to identify long and short DNA sequence alterations [see, for example, (Kawall et al., 2020; Chu and Agapito-Tenfen, 2022; Park et al., 2023)] that the unintended changes can be detected in the cell. By comparing the “wild type” with the one resulting from NGT applications, the unintended genetic alterations can become detectable and be made comparable to those that are likely to occur with conventional methods. As especially large deletions and chromosomal rearrangements are hardly detectable by standard short-range PCR based assays, it is important to combine multiple approaches to assess all types of gene alterations (Park et al., 2023). Park et al. (2023) state, no single tool can detect all types of large gene modifications accurately that can be caused by CRISPR/Cas9. Therefore, it is important to combine multiple approaches to comprehensively identify and assess the unintended changes throughout the genome [see also (Mou et al., 2017; Hahn and Nekrasov, 2019; Yasumoto and Muranaka, 2023)].

As DNA sequencing will not always allow the identification of the associated unintended biological effects, additional methods, such as transcriptomics and metabolomics, should be used to draw reliable conclusions [see (Kawall et al., 2020; EFSA et al., 2022c)]. If no unintended genetic alterations are detected that are specific to NGT processes, risk assessment may focus on the intended changes.

After comprehensive molecular characterisation has been concluded, further steps in risk assessment should follow, such as the analysis of plant composition, agronomic and other phenotypical characteristics, that also may include further investigations in regard to human health and the environment [see (EFSA, 2010; European Commission, 2013;)]. Data from the molecular assessment can be used to inform and guide these further steps in risk assessment and the development of a specific risk hypothesis.

## 5 Consequences of a comprehensive molecular characterisation and risk assessment regarding long-term cumulative effects

As cited above, Directive 2001/18/EC (European Parliament and Council of the European Union, 2001) also gives weight to cumulative long-term effects: “A general principle for environmental risk assessment is also that an analysis of the cumulative long-term effects relevant to the release and the placing on the market is to be carried out. “Cumulative long-term effects” refers to the accumulated effects of consents on human health and the environment, including *inter alia* flora and fauna, soil fertility, soil degradation of organic material, the feed/food chain, biological diversity, animal health and resistance problems in relation to antibiotics.” Furthermore, similarly to Commission Directive (EU) 2018/350 (European Commission, 2018), Commission Implementing Regulation (EU) No 503/2013 (European Commission, 2013) also requires the assessment of stacked events in regard to their “potential additive, synergistic or antagonistic effects resulting from the combination of the transformation events.”

It should not be overlooked that several databases show that there are dozens of current NGT projects using species such as oilseed rape (*Brassica napus*), tomato (*Solanum lycopersicum*) or wheat (*Triticum aestivum*) [for example see (Koller et al., 2023)]. In this respect, it is necessary to consider the overall gene pool of the species concerned. As Koller et al. (2023) show, if NGTs are used to generate different traits in one species, the resulting intended and/or unintended genetic changes may lead to interactions between the individual NGT organisms, and are thus relevant to risk assessment (Koller et al., 2023). There is also the need to take into account simultaneous spatial cultivation, further crossings and technical stacking of the various events. The resulting effects may be dependent on specific combinations of intended or unintended genetic variants, or the intended traits. In addition, the exposure to stress conditions in the receiving environment may have an influence. Even if all the individual events were considered to be “safe”, uncertainties or unknowns will still remain because of possible interactions of the intended and unintended genetic changes and associated effects in each event. The environmental risk assessment of individual events may, therefore, not be sufficient to predict and assess all these interactions. Special caution will be needed if the plants have the potential to persist, propagate and spontaneously cross in the environmental and/or perform gene flow to related species (Bauer-Panskus et al., 2020).

When developing relevant risk scenarios [see (Koller et al., 2023)], it also has to be considered that unintended genetic changes might be passed to offspring and introgress various genetic backgrounds that, for example, can cause changes in gene expression. Furthermore, the unintended genetic changes caused by NGT processes may also accumulate through subsequent crossings in following generations. This can result in phenotypes that differ significantly from those of their precursor plants (Bauer-Panskus et al., 2020).

As also mentioned by Koller et al. (2023), unpredictable genomic interactions may, for example, be caused by cryptic gene variants depending on the genetic background. Cryptic variations are considered to be mutations that, regardless of whether they occur naturally or are introduced by technical processes, have little or no phenotypic consequences unless exposed to additional genetic or environmental interactions, as for example discussed in the context of tomatoes (Rodríguez-Leal et al., 2017; Soyk et al., 2019; Alonge et al., 2020). Therefore, the genomic interactions emerging from spontaneous crossings or intended stacking may also become relevant to the assessment of unintended (as well as intended) genetic changes caused by NGT processes.

In some cases, too many uncertainties may remain due to the potential interactions and cumulative effects. Therefore, cut-off criteria will be needed to identify applications that will not allow robust conclusions on safety (Bauer-Panskus et al., 2020).

## 6 Discussion

As shown in this review, NGTs can cause different intended and unintended genetic changes in comparison to conventional breeding (including random mutagenesis). Relevant differences concern the site of the genetic alterations and their resulting pattern in the genome, the insertion of transgenes and the probability of chromothripsis-like events occurring at specific genomic sites.

It is conceivable that in some cases, the unintended genetic changes may have a higher relevance for risk assessment than the intended changes. Therefore, requirements regarding a mandatory investigation of intended and unintended genetic alterations, e.g. in the context of the EU GMO regulation, seem to be a scientifically justified necessity as also confirmed by Eckerstorfer et al. (2023).

There is an ongoing debate within the EU about the future regulation of NGT plants. Therefore, the European Food Safety Authority (EFSA), as mandated by the European Commission, has published several opinions dealing with aspects of risk assessment in relation to NGT plants (EFSA, 2012; EFSA, 2020; EFSA E. et al., 2021; EFSA, 2022c). As EFSA is a main source of science-based decision-making in the EU, we think it is important to compare our findings with the EFSA opinions.

EFSA concluded that in some cases, intended and unintended effects caused by NGT processes may require in-depth risk assessment. For example, EFSA (EFSA et al., 2021a) discusses an NGT wheat with a reduction of alpha-gliadin proteins (Sánchez-León et al., 2018). In this wheat, 35 out of 45 targeted alpha-gliadin genes were altered with CRISPR/Cas (SDN-1) to reduce the gluten content in food products. Many insertions and/or deletions at the targeted DNA sequences were described. EFSA came to the conclusion that the intended and unintended changes at the target sites pose in this case new challenges for risk assessment: “While plants with a small number of mutations have already reached the market, the large number of mutations required to achieve gluten-free wheat is far beyond any plant previously assessed. This is likely to require SynBio approaches to correctly identify all gliadins and glutenins in the hexaploid genome of bread wheat and to identify an engineering strategy that introduced mutations of the correct nature and positions in each gene to prevent the accumulation of any peptide fragments associated with initiation of the inflammatory cascade” (EFSA et al., 2021a).

From the findings of EFSA it seems that at least each targeted genetic site would undergo a detailed examination to determine whether the alpha-gliadin proteins are still produced, or if new proteins are being unintentionally produced, or if there are any other unintended effects.

Furthermore, EFSA (2020) also believes that the unintended insertions of transgenes in NGT plants need to be risk assessed: “When plant transformation is used to introduce the SDN module, the unintended insertion of plasmid DNA or other exogenous DNA into the plant genome can happen. Furthermore, the application of some methods (e.g. transient expression and DNA-free methods) to achieve SDN-1 and SDN-2 modifications can result in the unintended integration of exogenous DNA whose sequence may be known *a priori* [examples of unintended on-target insertion of exogenous DNA can be found in Clasen et al. (2015), Andersson et al., 2017, Norris et al. (2020), Solomon (2020)]. If the final product is not intended to retain any exogenous DNA, the applicant should assess the potential presence of a DNA sequence derived from the methods used to generate the SDN modification (e.g. plasmids or vectors). It should be noted that the assessment of the unintentional integration of exogenous DNA is already part of the molecular characterisation in the risk assessment of GM plants, under EU Regulations. Therefore, this is not to be considered a new requirement for risk-assessing genome-edited plants.” (EFSA, 2020).

However, in regard to other off-target effects, EFSA indicates that these would not require mandatory risk assessment, as they would be the same type of mutations caused by conventional breeding and/or random mutagenesis. A lot of emphasis is placed on the number of mutations–these are generally considered to be lower for NGTs in comparison to non-targeted methods. It appears to have escaped the notice of EFSA that these criteria may not be sufficient to draw reliable conclusions on health and environmental safety.

EFSA already dealt with the issue of unintended genetic changes in its opinion published in 2012. In its opinion, EFSA only addressed the type of mutations (such as indels) and the frequency of mutations. EFSA (2012) concluded at that time: “Whilst the SDN-3 technique can induce off-target changes in the genome of the recipient plant these would be fewer than those occurring with most mutagenesis techniques used in conventional breeding. Furthermore, where such changes occur they would be of the same types as those produced by conventional breeding techniques.”

EFSA in its 2020 opinion again deals with the frequency and type of mutations and does not consider other criteria, e.g. the site of the mutation, the genomic context, the resulting genetic combinations or any associated unintended phenotypical effects (EFSA, 2020). As EFSA (2020) states in its summary: “The EFSA Opinion on SDN-3 concluded that the application of SDN-3 can induce off-target mutations but these would be fewer than those occurring with most mutagenesis techniques (EFSA, 2020). Where they do occur, these changes would be the same types as those derived by conventional breeding techniques (EFSA, 2012). As SDN-1 and SDN-2 techniques use the same molecular mechanisms to generate DSB as SDN-3, the conclusions for SDN-3 are also applicable to SDN-1 and SDN-2.”

Once more, in its updated opinion on cisgenic plants, EFSA deals with the frequency and type of mutations and states that the frequency of mutations might be lower in the case of SDN-plants in comparison to previously used breeding methods (EFSA, 2022c). Again, EFSA did not consider the site of the mutation, the resulting gene combinations and specific unintended effects that may by caused by NGT processes. It appears that EFSA also became aware of some gaps in research, stating that: “Moreover, the GMO Panel was not mandated to provide a comprehensive literature review on the SDN-based technology and its unintended effects.” (EFSA, 2022b).

We conclude that the differences in the EFSA findings and our review are to a certain extent due to methodology: in regard to off-target unintended genetic changes resulting from NGT processes, EFSA mainly considered the overall frequency of mutations and the types of mutation that can be observed. However, EFSA did not take into account that unintended genetic changes caused by the processes of NGTs may not occur randomly across the genome and its biological effects may depend on the genomic regions that are targeted by the NGT processes. Therefore, EFSA did not consider the likelihood of unintended changes occurring at specific sites. It also did not consider resulting specific gene combinations, the frequency of chromothripsis-like events or the emergence of unintended gene products. Unintended effects in regard to the phenotypes and the environment were also not taken into consideration, although they may be associated with these unintended genetic changes.

## 7 Conclusion

As required in current EU regulation, unintended genetic changes and their potential effects have to be taken into account in the mandatory molecular characterisation and risk assessment of NGT plants. This requirement is relevant to the single event as well as all events within the gene pool of the species.

Since the unintended genetic changes as categorized above can neither be predicted nor excluded *a priori*, comprehensive molecular characterisation and risk assessment has to be performed for each single event. In many cases, if unintended genetic changes are caused by the processes of NGT, they may not occur randomly across the genome and its biological effects may depend on the genomic regions that are targeted by the NGT processes. Therefore, risk assessment should aim to identify those unintended genetic changes which (for example, in regard to the site, the frequency, its potential gene products or its origin) are unlikely to occur with conventional (non-regulated) methods. The methodology to identify these changes should include WGS by using long read sequencings, also in combination with other methods for gene analysis (Park et al., 2023). Comparison should be performed to the genome of the “wild type” plants that were used as starting point. In addition, comparisons with genome databases may be performed.

Furthermore, the comprehensive molecular characterisation and risk assessment should also comprise “Omics” (such as transcriptomics, proteomics and metabolomics) as discussed by EFSA (EFSA et al., 2022c).

It will depend on the findings of this molecular characterisation what further data for risk assessment will be required, i.a. for the analysis of plant composition and other phenotypical characteristics [as, for example, outlined in (EFSA, 2010; European Commission, 2013)].

Even if specific unintended effects arising from molecular changes due to NGTs cannot be identified in a specific event, the regulator still has to consider cumulative effects and potential interactions which could result from future crossings within the same species or wild relative species.

The resulting unintended effects may be dependent on specific combinations of intended or unintended genetic variants, which may become obvious only after exposure to stress conditions in the receiving environment (Koller et al., 2023).

If unintended genetic changes, potentially causing adverse effects are overlooked, these may endanger health, the environment and also agricultural production. Therefore, unintended genetic changes caused by the processes of NGTs has to be included in mandatory risk assessment before the plants are released into the environment or placed onto the market.
